# Different sleep pattern in over-weight/obese women with polycystic ovary syndrome

**DOI:** 10.3389/fendo.2023.1068045

**Published:** 2023-02-10

**Authors:** Emma Oberg, Liselotte Blomberg, Torbjörn Åkerstedt, Angelica Lindén Hirschberg

**Affiliations:** ^1^ Department of Women’s and Children’s Health, Division of Neonatology, Obstetrics and Gynecology, Karolinska Institutet, Stockholm, Sweden; ^2^ Department of Pelvic Cancer, Theme Cancer, Karolinska University Hospital, Stockholm, Sweden; ^3^ Department of Gynecology and Reproductive Medicine, Karolinska University Hospital, Stockholm, Sweden; ^4^ Department of Clinical Neuroscience, Karolinska Institutet, Stockholm, Sweden

**Keywords:** polycystic ovary syndrome (PCOS), sleep, behavior modification, lifestyle, actigraphy

## Abstract

**Context:**

Sleep duration and sleep quality have important health implications although our knowledge of objectively measured sleep variables in women with Polycystic Ovary Syndrome (PCOS) is limited.

**Objective:**

To compare sleep variables assessed by actigraphy in over-weight/obese women with PCOS and controls, and to assess sleep variables after behavioral modification intervention in comparison with minimal intervention in a randomized trial.

**Design:**

Randomized controlled trial, and a control group.

**Setting:**

Outpatient gynecological clinic at a university hospital in Sweden.

**Participants:**

39 women fulfilling all Rotterdam PCOS criteria, randomized to behavioral modification intervention or minimal intervention and 21 controls with no other metabolic disease, all aged 18‐40 years with a BMI ≥ 27 kg/m^2^.

**Intervention:**

A four-month behavioral modification intervention including weekly group meetings focusing on behavioral and healthy lifestyle aspects. Minimal intervention reflecting standard care.

**Main outcome measure:**

Sleep durations and sleep efficiency assessed by actigraphy.

**Results:**

Compared to the control group, women with PCOS had significantly shorter time in bed (501 vs 548 min, p= 0.049), sleep time over 24 hours (448 vs 567 min, p=0.005) and sleep time at night (434 vs 511 min, p=0.002), poorer sleep efficiency (87 vs 93%, p<0.001), and longer wakefulness after sleep onset (64 vs 38 min, p<0.001). However, total sleep time at night for women with PCOS (7.2hrs) was within the normal range. Following behavioral modification intervention, the reduction from baseline in sleep over 24 hours and in the daytime sleep were significant compared to the minimal intervention group (78 min, p=0.009 and 43 min, p=0.003 respectively).

**Conclusions:**

We found over-weight/obese women with PCOS to have normal sleep duration, but worse sleep efficiency than controls. Behavioral modification intervention seems to reduce the amount of daytime sleep, suggesting improved sleep behavior.

**Clinical trials registration:**

https://doi.org/10.1186/ISRCTN48947168, identifier ISRCTN48947168.

## Introduction

Polycystic ovary syndrome (PCOS) is the most common endocrine disorder in fertile women with a prevalence of 8-13% (1). It is characterized by oligo- or anovulation, clinical or biochemical hyperandrogenism and polycystic ovaries on ultrasound (2, 3). PCOS is a heterogenous condition and has previously been considered a disorder of infertility and clinical hyperandrogenism mainly manifested by hirsutism. Today, other aspects of PCOS are increasingly recognized including metabolic disturbances such as insulin resistance and abdominal obesity, as well as psychiatric morbidity e.g. depression and anxiety (4–8). We have recently demonstrated that psychological well-being is severely impacted in overweight women with PCOS (9).

In addition, sleep disturbances have been reported as common among women with PCOS, and the occurrence of obstructive sleep apnea (OSA) is higher than for controls (10–15). OSA is characterized by daytime sleepiness, somnolence and fatigue, restless sleep, and morning headaches (16). The most recent international PCOS clinical guidelines, recommend screening for OSA to identify and alleviate symptoms and to explore if fatigue potentially contributes to mood disorders as well as to attempt optimizing the sleep (2). Other aspects of sleep behavior than OSA in women with PCOS, such as sleep time, timings of sleep and sleep efficiency, have mainly been characterized by studies looking at self-reported parameters (10, 15, 17). Furthermore, evidence from objective measurements of sleep behavior in women with PCOS using actigraphy or polysomnography other than for diagnosing OSA is scarce (18, 19).

Studies on non-PCOS, including female only populations, have shown that short sleep duration is linked to obesity, decreased insulin sensitivity as well as increased hunger and appetite (20–22). In addition, daytime napping is positively correlated with body mass index (BMI) and waist circumference in women (23). Furthermore, sleep duration shorter than 6 hours (360 minutes) is associated with menstrual cycle disturbances (both long and short cycles) (24). There is also evidence showing that altering the timings of wakefulness and sleep, being awake at night and asleep during the day, is associated with impaired glucose tolerance and increased insulin resistance (21). Finally, both short and long sleep times are associated with increased mortality (25).

The observations in women with PCOS indicate a need for investigating if overweight/obese women with PCOS differ from body mass index (BMI)-matched controls with respect to objective indicators of sleep efficiency and awakenings, the timing of sleep, its variability, as well as sleep duration. Moreover, since lifestyle management including exercise, diet, behavioral intervention, or a combination of those, is the first line treatment for over-weight women with PCOS, it is important to assess its impact on sleep health variables in this population (2).

The primary objective of this study was therefore to assess objective sleep variables by actigraphy in over-weight/obese women with PCOS in comparison with BMI- and age-matched controls. In addition, to investigate the effect of behavioral modification intervention in comparison with minimal intervention on sleep variables in a randomized trial of women with PCOS, as well as to look for any correlations between sleep variables and anthropometric, endocrine and psychological well-being parameters.

## Materials and methods

### Study design

This is a secondary analysis of data from a Randomized Controlled Trial (RCT) (ISRCTN48947168), previously described in Oberg et al. (26) where 68 over-weight/obese women with PCOS where randomized to receive behavioral modification intervention or minimal intervention in a 1:1 ratio for four months with a further follow up at 12 months (26). Weight change and reproductive outcomes in relation to weight loss were the primary outcomes (26). Other previously published data include psychological well-being parameters as well as endocrine and metabolic variables (9).

In this study, we have focused on objectively measured sleep variables in the same population of over-weight/obese women with PCOS and measured the treatment effects of the four-month intervention within and between groups. Sleep-registration with actigraphy was initiated part of the way through the inclusion period resulting in 39 out of the 68 women with PCOS completing the sleep registration at baseline (22 in the behavioral modification intervention group and 17 in the minimal intervention group), and 28 women at four months (13 in the behavioral modification intervention group and 15 in the minimal intervention group). In addition, we have introduced a control group of 21 women with comparable BMI and age to the women with PCOS without any metabolic-, endocrine or other illness, and compared the baseline sleep variables from our whole population of over-weight/obese women with PCOS (n=39) with the controls. Written consent has been obtained from all study participants. The local ethics committee in Stockholm has approved the study (2012/146-31/3, 2012/1762-32, 2014/1406-32, 2020/00653).

### Women with PCOS

Study participants were recruited through adverts in a local newspaper as well as on a website for clinical studies. Inclusion criteria were BMI ≥ 27 kg/m^2^, age between 18-40 years and using the PCOS Rotterdam consensus, fulfilling all three PCOS diagnostic criteria: oligo- or anovulation, clinical or biochemical hyperandrogenism and polycystic ovaries on ultrasound (3). A serum testosterone > 310 pg/mL measured by tandem mass spectrometry indicated biochemical hyperandrogenism, and a Ferriman-Gallwey score ≥ 8 was used to define hirsutism and a measure of clinical hyperandrogenism (27). Ovaries were considered polycystic if at least one had a volume ≥ 10 mL or ≥ 12 antral follicles when using transvaginal ultrasound (Sonoline SI-250, Siemens Healthcare Diagnostics) (2, 3). Criteria of exclusion were other chronic illnesses including eating disorders or ongoing medication, pregnancy or breast-feeding, smoking, or a substantial change in weight during the past year as well as working night-shifts. A washout period of three months was used for participants using a hormonal contraceptive.

### Controls

The women used as controls were recruited *via* a web-site advert based on the same inclusion criteria of age (18 to 40 years old) and BMI (≥ 27 kg/m^2^) as the women with PCOS. The controls also needed to have a regular menstrual cycle length of 23 to 32 days. Exclusion criteria, in addition to the ones described above for the patient group, were having oligo- or amenorrhea, clinical or biochemical hyperandrogenism or a previous diagnosis of PCOS.

### Study intervention

#### Behavioral modification intervention

We used a behavioral modification intervention, developed as a training course with focus on achieving long-term weight control (26). The study participants were divided into small groups in which they attended weekly meetings throughout the four-month intervention period together with the course leader working through topics such as personal leadership, mindfulness, problem solving, stress management, stimulus control, techniques for avoiding instant gratification as well as more practical aspects concerning weight control, diet and physical activity. Preparation for each meeting by reading and personal reflection was encouraged (28). In addition, individual coaching sessions with the course leader were held once a month.

#### Minimal intervention

The minimal intervention group received standard care comprising oral and written information on healthy living including advice on diet and exercise delivered by a research midwife.

### Procedures

#### Women with PCOS

Before and after the 4-month intervention, the women with PCOS underwent a physical examination on menstrual cycle day 6-8. In women with oligo- or amenorrhea, a bleeding was induced by taking 10 mg medroxyprogesterone for seven days. A thorough medical history was taken, anthropometric measurements were obtained, a gynecological examination including transvaginal ultrasound was completed and fasting blood sampling was carried out allowing for analysis of hormones, binding proteins and metabolic variables. The participants also filled in a questionnaire assessing the psychological general wellbeing index (PGWBI) at baseline and 4 months.

#### Controls

A medical history was taken from the controls to ensure they fulfilled the entry criteria and no exclusion criteria. Fasting blood sampling was carried out on cycle day 6-8 to allow for analysis of hormones and binding proteins and anthropometric measurements were obtained. The controls did not receive any intervention and were only assessed at baseline.

### Actigraph assessment of sleep

Both the women with PCOS and the controls wore an actigraph, ActiSleep+ (ActiGraph) device on their non-dominant wrist or in some cases where this was not possible, around their ankle. They were encouraged to wear the device for 7 consecutive days, the whole time apart from when showering/taking a bath or undertaking other water-based activities. Patients and controls with recordings for less than three consecutive periods of 24 hours were excluded from the study. The PCOS study population carried the actigraph at baseline and at 4 months, and the control group only at baseline.

Actigraphy uses an accelerometer that measures movements and uses three-dimension acceleration data which are converted to estimated sleep parameters using the commonly used and validated Sadeh sleep algorithm (29). A recent study validating actigraphy as a method of assessing sleep variables compared to the gold standard method for sleep assessment of polysomnography, found actigraphy to give valid estimates of total sleep time, wake after sleep onset as well as sleep efficiency (30). The manufacturer’s data analysis software ActiLife 6 (ActilGraph) was used to extract data from the devices and each patient’s report was reviewed manually by the first author (EO). Recordings of sleep durations were: total sleep time over 24h (TST 24h, min), time in bed at night (TIB, min), total sleep time at night (TST night, min) and total sleep time during the day (TST day, min) (defined as a new episode of sleep initiated 30 minutes or later after time of rising, when occurring after 08:00). In addition, the time of rising (ToR) and the bed time were recorded. The number of wakeups (n) and wakefulness after sleep onset (WASO, min), as well as energy expenditure (kcal/day) and steps taken (n) were extracted. The sleep parameters for all recorded consecutive 24-hour periods obtained from the actigraph were averaged for each participant. Averages for weekdays (Monday to Thursday) and weekends (Friday and Saturday) were also obtained for all sleep parameters. Sleep efficiency (TST night/TIB x100) was calculated.

### Biochemical measurements

The sex steroids were analyzed by liquid chromatography tandem-mass spectrometry (31, 32). The analyses of binding proteins and other hormones were carried out using electrochemiluminescence immunoassay (ECLIA) from Roche Diagnostics AG (CH 6343 Rotkreuz, Switzerland) (Cobas8000^®^) the Department of Clinical Chemistry, Karolinska University Hospital, Stockholm, Sweden. Free androgen index (FAI) was calculated (testosterone nmol/L divided by SHBG nmol/L x 100).

### Assessment of psychological general well-being

The non-disease specific questionnaire PGWBI was used to assess the psychological well-being. The PGWBI contains 22 questions with 6 answers to choose from and it assesses the well-being during the previous month (33, 34). The questions are divided into the six dimensions of *anxiety, depressed mood*, *positive well-being*, *self-control*, *general health* and *vitality*. All dimensions can be added to achieve a *global score* (34). Greater well-being is always indicated by a higher score (e.g. a high score for depressed mood indicates a greater well-being) (34). We have previously shown that the well-being in this group was very low at baseline and that some aspects of well-being (anxiety, general health and depressed mood) improved following behavioral modification (9).

### Statistics

**Figure 1:**
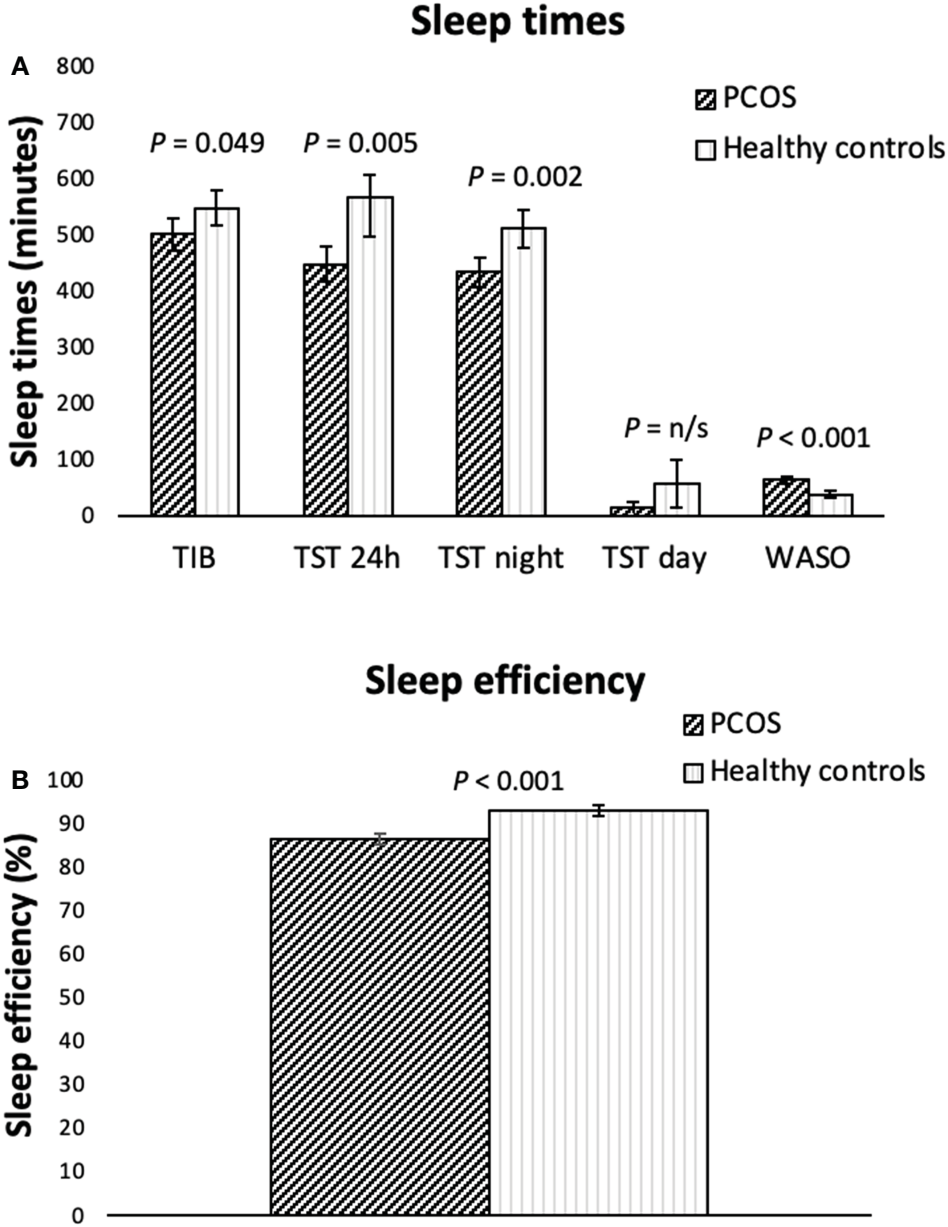
Actigraphy measured sleep variables (**A**, sleep times, **B**, sleep efficiency) for overweight/obese women with PCOS (n=39) and healthy controls (n=21) of a similar age and weight presented as means including the 95% CI. P-values are adjusted: controlling for being in a relationship, having children, BMI and waist circumference as covariates. H, hours; min, minutes; TIB, time in bed; TST, total sleep time; WASO, wakefulness after sleep onset. Sleep efficiency: total sleep time/time in bed x 100.

**Figure 2:**
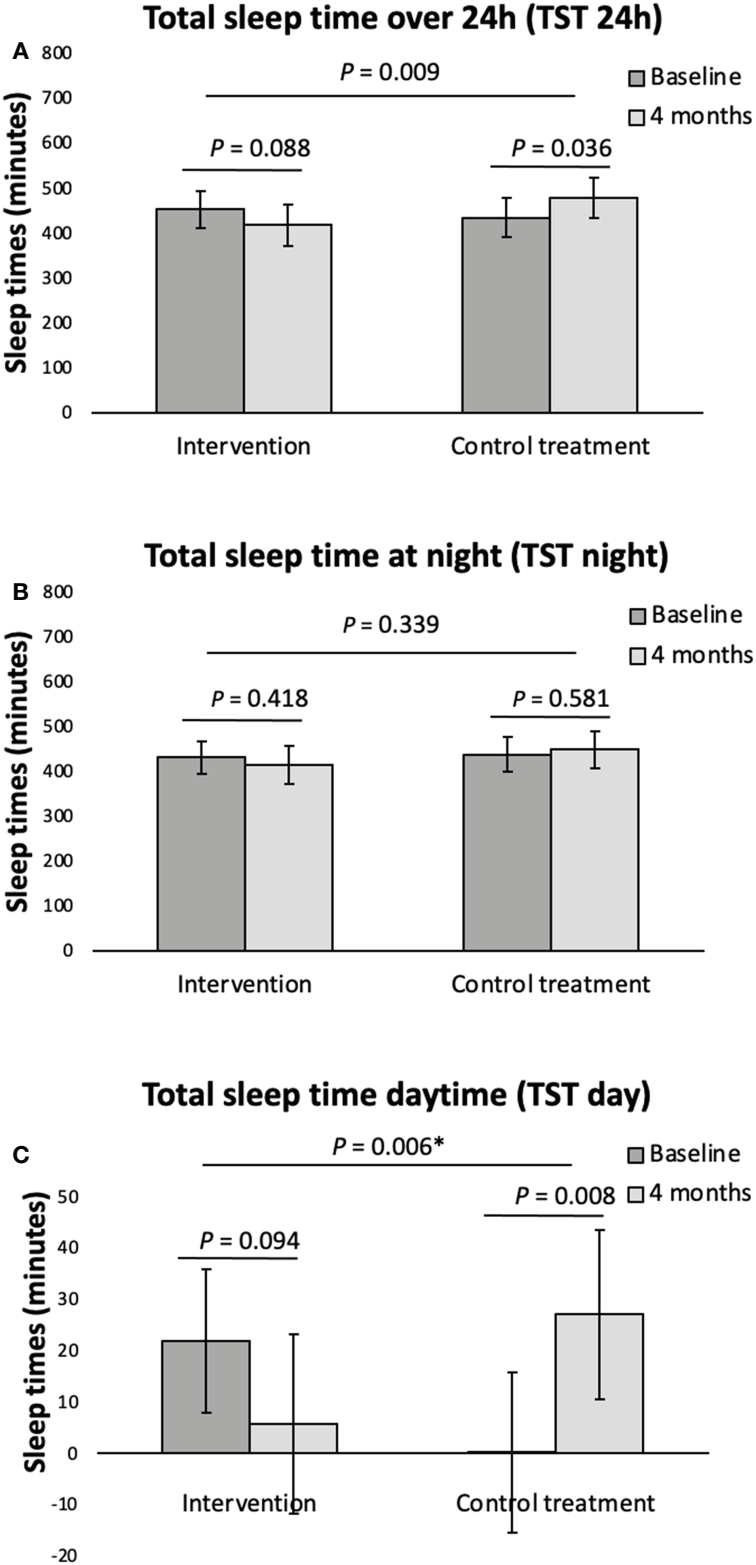
Actigraphy registered sleep variables (**A**, total sleep time over 24h, **B**, total sleep time at night, **C**, total sleep time daytime) at baseline and following 4 months intervention for the overweight women with PCOS. Data given as mean with its 95% Confidence Interval calculated using a mixed model on an ITT basis. *P-value corrected for the confounder TST day using analysis of covariance. TST, total sleep time.

Statistical analysis was carried out using SPSS software version 26 (IBM; Stockholm, Sweden). In **Tables 1**, **2**, proportions or percentages are used to present categorical data, continuous data is presented as means ± standard error (SE). For continuous data, normal distribution was tested for using histograms, as well as calculation of skewness. Student’s t-test was used to assess the difference in baseline characteristics between the groups for continuous variables, and Fisher’s Exact test for categorical data. Analysis of covariance was used to compare the group of women with PCOS with the controls with respect to the sleep variables in **Figure 1**, adjusted for the potential confounders: being in a stable relationship, having children, BMI and waist circumference. A linear mixed model analysis was used to assess the effects of the intervention within and between the treatment groups at four months (**Table 2** and **Figure 2**) where the factors used were treatment (behavioral modification intervention and minimal intervention), time (baseline and four months) and the interaction treatment x time. The results from the mixed model analysis are presented as means as well as SE for within group analysis and as the mean difference of the change from baseline along with the 95% confidence intervals (CI) for between group analysis. The analysis was carried out on an intention-to treat (ITT) basis. All analyses were carried out both on the mean of all 24-hour sleep periods recorded for each participant, as well as the mean of the weekday (Monday to Thursday) and weekend (Friday and Saturday) sleep. The bivariate correlation analysis of baseline demographic, anthropometric, accelerometer data and endocrine variables with sleep parameters, as well as the correlation analysis between the change of these variables following the 4-month intervention period were performed using Spearman rank correlation. Statistical significance was assumed at *P*-values < 0.05. This is a secondary analysis, where the power calculation based on weight change following intervention has previously been published (26).

**Table 1:** Baseline characteristics of the PCOS population as well as the control group.

	PCOS population (n=39) #	Controls (n=21)	*p*-value
Demographics			
Age (year)	30.1 ± 5.3	29.9 ± 5.2	0.849
Education			0.197
Primary	1/39 (2.6%)	0/21 (0%)	
Secondary	13/39 (33.3%)	3/21 (14.3%)	
University	14/39 (35.9%)	10/21 (47.6%)	
Current student	8/39 (20.5%)	8/21 (38.1%)	0.220
Unemployed	0/39	0/21	
In a stable relationship	26/39 (66.7%)	8/21 (38.1%)	**0.028**
Have children	16/39 (41.0%)	3/21 (14.3%)	**0.044**
Anthropometric			
Body weight (kg)	93.2 ± 16.2	86.6 ± 10.7	0.083
BMI (kg/m^2^)	34.1 ± 33.9	31.6 ± 31.6	0.064
Waist circumference (cm)	103.7 ± 11.3	94.4 ± 8.4	**0.001**
Hip circumference (cm)	116.6 ± 10.5	117.4 ± 7.9	0.813
WHR	0.89 ± 0.06	0.81 ± 0.05	**< 0.001**
Endocrine Variables			
FSH (IU/L)	6.6 ± 3.5	7.1 ± 2.1	0.584
LH (IU/L)	6.7 ± 3.2	6.5 ± 2.9	0.887
Testosterone (pg/mL)	392.5 ± 137.9	270.0 ± 95.2	**0.001**
SHBG (nmol/L)	27.3 ± 15.1	54.8 ± 40.1	**< 0.001**
Free Androgen Index	6.5 ± 3.9	2.3 ± 1.0	**< 0.001**
Androstenedione (pg/mL)	1628 ± 525	1055 ± 251	**< 0.001**
Estradiol (pg/mL)	49.9 ± 26.7	48.9 ± 32.3	0.906

• Baseline categorical data is presented as a proportion/percentage, and continuous data as means ± standard deviation.

• To determine the difference between groups, the independent sample t-test was used for continuous data and the Fisher’s exact test for categorical data.

• BMI, body mass index; FSH, follicle stimulating hormone; LH, luteinizing hormone; min minutes; SHBG, sex hormone-binding globulin; WHR, waist/hip ratio.

• Free androgen index calculated as testosterone nmol/L divided by SHBG nmol/L x 100.

• # subset of a larger population where data previously has been published.

• P-values in bold indicate statistical significance.

**Table 2:** Sleep, anthropometric, endocrine and metabolic parameters at baseline and after 4 months of intervention for the women with PCOS.

	Behavioral Modification Intervention (n=22)			Minimal Intervention (n=17)			Difference in change between groups	
Sleep parameters	Baseline	4 months	*P*-value	Baseline	4 months	*P*-value	Behavioral Modification - Minimal Intervention	*P*-value
TIB (min)	497.2 (459-535)	464.0 (416-512)	.208	502.4 (460-545)	509.7 (465-555)	.783	-40.5 (-115.7 to 34.7)	.280
Sleep efficiency (%)	86.8 (85.1-88.5)	87.0 (85.0-89.1)	.803	86.8 (84.9-88.7)	87.4 85.4-89.5)	.534	-0.40 (-3.4 to 2.5)	.786
Nb wakeups/night	18.4 (15.6-21.1)	19.1 (6.1-32.1)	.902	17.5 (14.4-20.5)	24.6 (12.5-36.8)	.221	-6.4 (-24.0 to 11.2)	.445
WASO (min)	63.1 (54.0-72.1)	58.8 (47.4-70.2)	.483	62.9 (52.7-73.1)	62.7 (51.9-73.4)	.964	-4.0 (-21.3 to 13.4)	.646
Anthropometric #								
Body weight (kg)	91.9 (84.6-99.2)	89.4 (81.9-96.8)	**.042**	97.7 (90.1-105)	95.5 (87.8-103)	.087	-0.3 (-3.9 to 3.2)	.842
BMI (kg/m^2^)	33.4 (31.2-35.5)	32.4 (30.2-34.6)	**.037**	35.6 (33.3-37.9)	34.8 (32.5-37.1)	.077	-0.1 (-1.4 to 1.1)	.841
Waist (cm)	103.5 (98.4-109)	99.9 (94.6-105)	**.023**	106.0 (101-111)	102.4 (96.9-108)	**.036**	0.003 (-4.5 to 4.5)	.999
Endocrine variables #								
Testosterone (pg/mL)	360.1 (302-418)	370.7 (301-441)	.759	434.9 (369-501)	361.8 (290-434)	.065	83.7 (-21.5 to 189.0)	.114
Free Androgen Index	5.5 (4.1-7.0)	4.8 (2.9-6.7)	.489	7.7 (6.0-9.4)	5.5 (3.5-7.3)	.060	1.5 (-1.7 to 4.8)	.349
Metabolic variables #								
Fasting Insulin (mIE/L)	13.5 (9.2-17.9)	11.3 (6.3-16.4)	.324	17.0 (12.1-21.8)	17.5 (12.3-22.7)	.819	-2.8 (-9.4 to 3.9)	.401
Fasting Glucose (mmol/L)	4.7 (4.4-5.0)	4.7 (4.3-5.0)	.751	4.9 (4.5-5.2)	5.0 (4.7-5.4)	.238	-0.2 (-0.7 to 0.2)	.280
HOMA	2.9 (1.8-4.1)	2.4 (1.0-3.7)	.291	3.8 (2.6-5.1)	4.3 (3.0-5.7)	.401	-1.0 (-2.6 to 0.6)	.185

• Data given mean and mean differences with its 95% Confidence Interval.

• BMI, body mass index; HOMA, Homeostatic Model of Assessment; min, minutes; Nb, number; TIB, time in bed; WASO, wakefulness after sleep onset.

• Free androgen index calculated as testosterone nmol/L divided by SHBG nmol/L x 100.

• # subset of a larger population where data previously has been published.

• Homeostatic Model of Assessment (HOMA) index calculated using the formula (insulin mIE/L) x (glucose mg/dL)/405.

• P-values in bold indicate statistical significance.

## Results

### Baseline characteristics

**Table 1** outlines the baseline demographic, anthropometric, and endocrine variables for the whole group of women with PCOS (behavioral modification intervention and minimal intervention), as well as for the control group. The whole group of women with PCOS and the controls were comparable in regard to age and BMI on a group level. The women with PCOS had a larger waist circumference and a higher WHR. Furthermore, there were expected between group differences for the endocrine variables, with the women with PCOS having higher androgen levels than the controls. The percentage of women with PCOS with biochemical hyperandrogenism was 76%. In addition, the women with PCOS were more likely to be in a stable relationship and to have children than the controls. There was no difference in education level or in the number of current students between the groups. None of the study participants was unemployed. There was no difference in baseline characteristics between the women with PCOS randomized to behavioral modification intervention and minimal intervention as previously published (9, 26).

### Sleep in women with PCOS compared to controls

When looking at all days of the week, the women with PCOS had significantly shorter mean total sleep time over 24 hours (TST 24h, min), time in bed at night (TIB, min), total sleep time at night (TST night, min), total sleep time during the day (TST day, min), poorer sleep efficiency (%), and longer periods of wakefulness after sleep onset (WASO, min) than the control group as shown in **Figures 1A, B**. However, we found no differences between the groups in the number of awakenings per night, or in the bed-time. The mean time of rising (ToR) occurred earlier for the PCOS patients than for the controls. When adjusting for being in a relationship, having children, BMI and waist circumference, the differences between groups for TST day and ToR disappeared, but all other differences between groups remained. When looking at the sleep parameters for weekdays only (Monday to Thursday) all differences between groups remained, apart from the TIB, where the difference no longer was significant (data not shown). Regarding sleep data for weekends only (Fridays and Saturdays), the women with PCOS still had a significantly shorter TST 24h (*p*=0.041), TST night (*p*=0.015), poorer sleep efficiency (*p*<.001) and a longer WASO (*p*<0.001) compared to controls.

### Sleep following behavioral modification intervention in women with PCOS

For the women with PCOS, the TST 24h and the TST daytime decreased in the behavioral modification intervention group albeit not significantly, following the 4-months behavioral intervention program, and increased in the PCOS minimal intervention group during the same period, as shown in **Figure 2**. This resulted in a significant difference following intervention in the TST 24h and the TST daytime between the PCOS women having received behavioral modification intervention and the minimal intervention group (**Figure 2**). As outlined in **Table 2**, we found no other within or between group differences to the actigraphy recorded sleep parameters following the 4 months behavioral modification intervention. No significant within or between group differences following intervention was seen when analyzing the data separately for weekdays (Monday to Thursday) and weekend (Friday and Saturday) (data not shown).

### Correlations between sleep variables and baseline characteristics

For the women with PCOS at baseline, there were no correlations between the objective sleep variables and the hormonal and anthropometric measurements respectively, nor were there any correlations between the changes in these variables following intervention. In the same study, we have previously shown that psychological well-being was severely reduced at baseline however behavioral modification intervention had a positive impact on some of the dimensions of well-being (9). In terms of correlations between sleep variables and the psychological well-being measurements at baseline, there was a positive correlation between sleep efficiency and the variables self-control (r = 0.41, *p* = 0.023) and a negative correlation between WASO and self-control (r = -0.38. *p* = 0.035).

## Discussion

To our knowledge, this is the first study investigating objectively measured sleep health variables in overweight women with PCOS both compared to controls, as well as after lifestyle intervention. We found that women with PCOS had poorer sleep efficiency and longer wakefulness after sleep onset than controls. Furthermore, there was a non-significant reduction in total sleep time over 24 hours, as well as in total daytime sleep following behavioral modification intervention, although this was significant when compared to the minimal intervention group. There was no reduction in the total sleep time at night, suggesting less daytime napping after behavioral modification intervention.

In adults, the recommended sleep duration is between 6h to 8h per night, where sleep over 9h could be used to detect co-morbidity (35). The women with PCOS had a mean total sleep time at night of 7.2h, whereas the control group had a somewhat longer night sleep of 8.5h. When looking at the sleep time over the 24-hour window, we found that the control group slept two hours longer than the PCOS women. This was partly due to 47 minutes longer time in bed, largely caused by later rising, as well as 57 minutes of daytime sleep (napping). Despite the longer time in bed, which normally results in less effective sleep, the sleep efficiency was significantly higher in the control group and wakefulness after sleep onset significantly shorter. Both are key indicators of sleep quality (36). This implies that sleep quality was better in the control group, despite their total sleep time being longer.

An increased sleep duration could have been an artefact of a wider sleep window. The data for the control group was partly recorded during the beginning of the COVID-19 pandemic, when working from home was recommended, which could have permitted a larger night time sleep window for this group as well as opportunities for daytime napping. In addition, the proportion of women with children was lower among the controls, and fewer were in a stable relationship. However, there was no difference in the proportion of students between the groups and no-one was unemployed, factors that could otherwise have permitted a wider sleep window and provided an explanation for the longer sleep times in the control group. When controlling for the potential covariates of being in a relationship, having children, BMI and waist circumference, the difference between groups in daytime sleep and time of rising disappeared, leading us to believe that being in a relationship and having children affected these variables. However, this does not alter the fact that sleep quality, in terms of sleep efficiency, was considerably higher in the control group even after adjusting for covariates.

Our findings are in agreement with two other studies that have found lower actigraphy recorded sleep efficiency in women with PCOS compared to controls; Shreeve et al. (19) looking at adults and Simon et al. (18) studying adolescents (18, 19). Another study using polysomnography (one night only in a sleep laboratory) by Sousa et al. (37), found lower sleep efficiency in a PCOS adolescent population compared to controls (37). Three other relatively small polysomnography studies based on adult PCOS populations vs healthy controls, Hachul et al. (38), Suri et al. (39) and Fogel et al. (11), also found poorer sleep efficiency in the PCOS groups but the results did not reach significance, potentially because of the small sample size (11, 38, 39). In terms of wakefulness after sleep onset, results from polysomnography studies by Sousa et al. (37), and Suri et al. (39) agree with our results of women with PCOS having a longer wakefulness after sleep onset than controls, however Simon et al. (18) and Hachul et al. (38) found no difference between the groups.

In terms of psychiatric well-being, we found a positive correlation between self-control and sleep efficiency and a negative correlation between self-control and wakefulness after sleep onset which is reasonable since the latter contributes largely to the sleep efficiency measure. A recent study by Yang et al. (40) found an association between self-reported sleep parameters and anxiety/depression status in Chinese women with PCOS, hypothesizing that sleep disturbances might be part of the etiology of the psychiatric co-morbidity in this patient group. However, the causality of these associations has to be investigated further (40).

In addition to comparing the objectively measured sleep variables in women with POCS with controls, we have also for the first time investigated the effects of lifestyle intervention on sleep variables in women with PCOS. We found that following behavioral modification intervention the overweight/obese women with PCOS displayed a non-significant reduction in the total sleep time over 24 hours and in daytime sleep, compared to the minimal intervention group where the total sleep time over 24 hours and daytime sleep increased, and the difference in these changes following intervention between groups was significant. As no such significant difference between groups was seen for the total sleep time at night, the reduction of total sleep time over 24hours is explained by less daytime napping following behavioral modification intervention. A meta-analysis based on a non-PCOS population by Zhong et al. (41) showed that daytime napping is associated with increased risk of death of all causes (41). Another meta-analysis found that daytime napping is associated with an increased risk of type 2 diabetes mellitus (42). This is in line with our previously published findings for the same population of women with PCOS showing improved metabolic parameters following behavioral modification intervention (26).

A strength of the study is the use of actigraphy for objectively measured sleep variables compared to the majority of studies on similar populations using subjective reporting of sleep quality. Actigraphy also allows for a non-invasive method of assessing sleep for a long period of time in a patient’s normal environment. However, actigraphy cannot be used for the assessment of obstructive sleep apnea. One limitation of his study is that the collection of sleep data for the control group partly was completed during the COVID-19 pandemic, when working from home was recommended by the authorities, enabling a wider window of sleep. In addition, there were differences in the proportion of women with children, as well as those in a stable relationship between the groups, however these differences were adjusted for in the statistical analyses.

In conclusion, we found that women with PCOS had shorter sleep duration, although within the normal range, but poorer sleep-efficiency and longer periods of wakefulness after sleep onset compared to controls. In terms of sleep quality after lifestyle intervention in the women with PCOS, there was a non-significant reduction in the total sleep time over 24 hours, as well as total daytime sleep following behavioral modification intervention, which was significant when compared to the minimal intervention group. Behavioral modification intervention seems to reduce the amount of daytime sleep, suggesting improved sleep behavior.

## Data availability statement

The raw data supporting the conclusions of this article will be made available by the authors, without undue reservation.

## Ethics statement

The studies involving human participants were reviewed and approved by Regionala etikprövningsnämnden Stockholm, Avdelning 3. The patients/participants provided their written informed consent to participate in this study.

## Author contributions

All authors contributed to the study conception and design. Data collection and analysis were performed by EO and ALH. The first draft of the manuscript was written by EO and all authors commented on previous versions of the manuscript. All authors contributed to the article and approved the submitted version.
